# Prediction of the potential geographic distribution of the ectomycorrhizal mushroom *Tricholoma matsutake* under multiple climate change scenarios

**DOI:** 10.1038/srep46221

**Published:** 2017-04-10

**Authors:** Yanlong Guo, Xin Li, Zefang Zhao, Haiyan Wei, Bei Gao, Wei Gu

**Affiliations:** 1 Northwest Institute of Eco-Environment and Resources, Chinese Academy of Sciences, Lanzhou 730000, China; 2 University of Chinese Academy of Sciences, Beijing 100049, China; 3 CAS Center for Excellence in Tibetan Plateau Earth Sciences, Beijing 100101, China; 4 College of Tourism and Environment, Shaanxi Normal University, Xian 710119, China; 5 College of Life Sciences, Shaanxi Normal University, Xian 710119, China

## Abstract

Effective conservation and utilization strategies for natural biological resources require a clear understanding of the geographic distribution of the target species. *Tricholoma matsutake* is an ectomycorrhizal (ECM) mushroom with high ecological and economic value. In this study, the potential geographic distribution of *T. matsutake* under current conditions in China was simulated using MaxEnt software based on species presence data and 24 environmental variables. The future distributions of *T. matsutake* in the 2050s and 2070s were also projected under the RCP 8.5, RCP 6, RCP 4.5 and RCP 2.6 climate change emission scenarios described in the Special Report on Emissions Scenarios (SRES) by the Intergovernmental Panel on Climate Change (IPCC). The areas of marginally suitable, suitable and highly suitable habitats for *T. matsutake* in China were approximately 0.22 × 10^6^ km^2^, 0.14 × 10^6^ km^2^, and 0.11 × 10^6^ km^2^, respectively. The model simulations indicated that the area of marginally suitable habitats would undergo a relatively small change under all four climate change scenarios; however, suitable habitats would significantly decrease, and highly suitable habitat would nearly disappear. Our results will be influential in the future ecological conservation and management of *T. matsutake* and can be used as a reference for studies on other ectomycorrhizal mushroom species.

Climate influences the growth and reproduction of species and thus is a determining factor in the distribution of species^1^,^2^,^3^. Climate at least partially controls the structure and function of essential terrestrial ecosystems^4^,^5^. Thus, future changes in climate are likely to induce biodiversity loss and may lead to changes in habitat distribution, habitat fragmentation, and an increased risk of extinction of endangered plants^6^,^7^,^8^. Empirical evidence demonstrates that plant and animal species have responded to recent climatic change by shifting their distributions poleward and to higher elevations^2^,^3^,^9^,^10^. These range shifts are likely to continue in many regions, resulting in range expansion or contraction or even extinction for some species^11^,^12^. Although climate change is a global phenomenon, regional and local climate change are greater concerns for rare plants^8^,^13^,^14^. Over the past few decades, many studies have focused on the effect climate change has on the spatial distribution of plant species, but few studies have examined the regional distribution of endangered ectomycorrhizal mushrooms^15^,^16^, and even fewer have considered soil and vegetation factors.

Fungi are essential components of all natural terrestrial ecosystems. Many ectomycorrhizal mushrooms play pivotal roles in the human diet and in agriculture, biotechnology, and forestry^15^,^17^. *Tricholoma matsutake* is an ectomycorrhizal fungus associated with Pinaceae and Fagaceae trees in China, Korea, Finland, the United States and Japan^18^. In China, suitable habitats for *T. matsutake* are mainly located in Yunnan Province, Sichuan Province, the Tibet Autonomous Region, Guizhou Province, Heilongjiang Province and Jilin Province. Due to its medicinal effects and attractive flavor, *T. matsutake* is one of the most economically important ectomycorrhizal mushrooms in Asia, particularly in China^17^,^19^. Modern pharmacology has confirmed that the polysaccharides and terpenoids extracted from the fruiting body of *T. matsutake* have antitumor and antioxidant properties^20^,^21^. In the wild, the growth of *T. matsutake* is extremely slow and limited to strict environmental conditions. *T. matsutake* grows only in high crown-density primeval forests without pollution, and thus the populations of this species are limited. The influence of ecological factors, such as temperature, moisture, soil properties, and terrain, on the development of the *T. matsutake* fruiting body remains unclear^18^,^21^. Since artificial cultivation is unavailable, natural populations of this species are under increasing threat, primarily from habitat disturbance and destruction^22^,^23^. Thus, determining the potential geographic distribution of this species and predicting how climate change would affect its geographic range are necessary and meaningful.

Models are essential tools to assess the potential responses of plants to climate change, particularly if large spatial and temporal scales are considered. Statistical species distribution models (SDMs) are increasingly used to forecast the potential changes in species distributions under different climate change scenarios^13^,^16^,^24^,^25^,^26^. By building a correlation between species existence and climatic and geographical features, SDMs can predict the current and future distributions of target species^24^. Over the past two decades, numerous SDMs from a range of methods, such as genetic algorithm for rule-set production (GARP)^27^, random forest (RF)^28^, ecological niche factor analysis (ENFA)^29^, artificial neural network (ANN)^30^, and maximum entropy (MaxEnt)^31^, have been developed. The prediction accuracy of MaxEnt is stable and reliable, even with incomplete data and small sample sizes^16^,^31^,^32^,^33^. In addition, MaxEnt requires only species presence data, and both continuous and categorical environmental data can be used as input variables.

In this study, species presence data at 91 locations and 24 climatic and environmental variables were analyzed using MaxEnt to predict the distribution of *T. matsutake* in China. MaxEnt was also used to simulate the potential habitat of *T. matsutake* under four climate warming scenarios (RCP 8.5, RCP 6, RCP 4.5 and RCP 2.6, given by the Intergovernmental Panel on Climate Change (IPCC))^34^. The objective was to select key environmental variables that are highly correlated with *T. matsutake* distribution and to predict the impact of climate change on the potential habitat of *T. matsutake*. Our results will supply advice for the protection and sustainable utilization of *T. matsutake* resources and establishing measures and proposals for the prediction of the distribution of ectomycorrhizal mushrooms under different climate change conditions.

## Results

### Distribution of suitable habitats in the current climate environment

**Figure 1:**
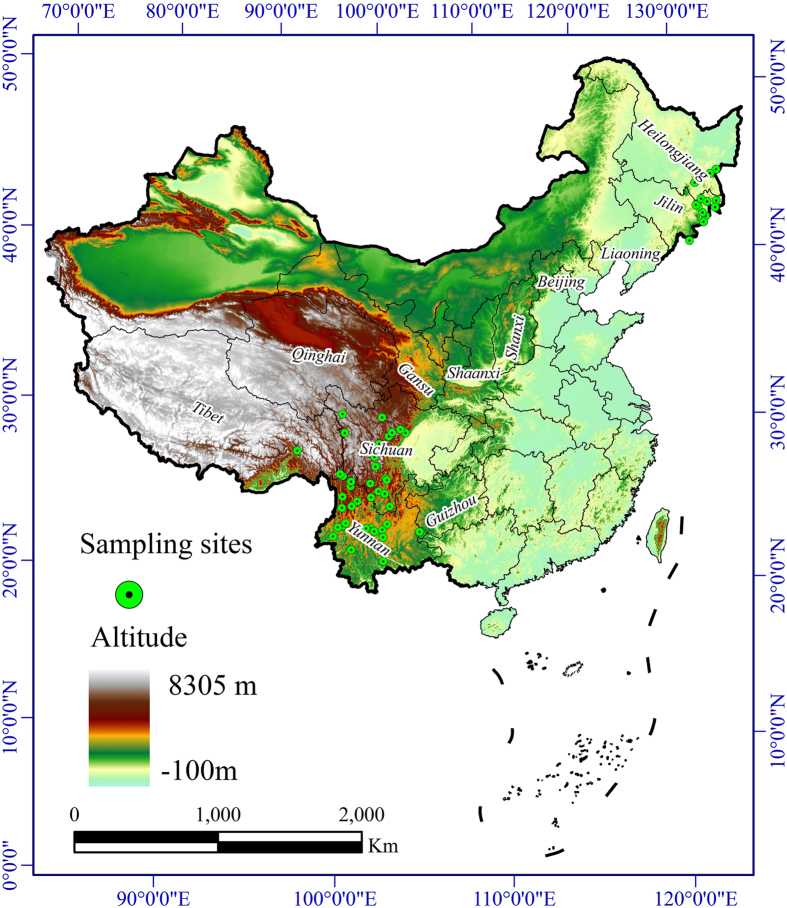
Geographic locations of *Tricholoma matsutake* populations in China. The map was plotted using ArcGIS 9.3 (ESRI, Redlands, CA, USA, http://www.esri.com/).

Data for a total of 91 present locations of *T. matsutake* were selected to build the model (Fig. 1). Using 19 bioclimatic variables and 3 topographic variables, MaxEnt provided satisfactory results, and the area under the receiver operating characteristic curve (AUC) values of the training and test data sets were 0.976 and 0.961, respectively. The AUC value of the MaxEnt model that used only the soil type variables was 0.925 for the training data set and 0.803 for the test data set. Thus, the model results can be considered satisfactory.

**Figure 2:**
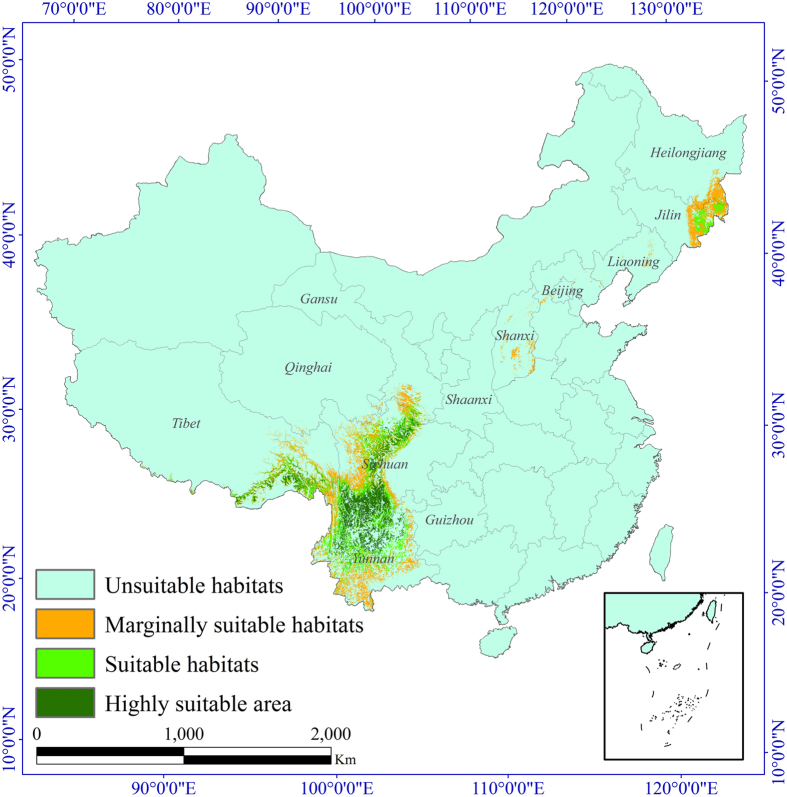
Predicted potential distribution of *Tricholoma matsutake* in China. The map was plotted using ArcGIS 9.3 (ESRI, Redlands, CA, USA, http://www.esri.com/).

The model results indicated two main regions of *T. matsutake* distribution (Fig. 2): northeastern China, which includes Heilongjiang and Jilin provinces, and southwestern China, which includes Yunnan Province, Sichuan province, the Tibet Autonomous Region, and Guizhou province. The calculated areas of the marginally suitable, suitable, and highly suitable habitat of *T. matsutake* in China are approximately 0.22 × 10^6^ km^2^, 0.14 × 10^6^ km^2^, and 0.11 × 10^6^ km^2^, respectively. The suitable and highly suitable habitats are mainly located in southeastern Heilongjiang, northeastern Jilin, central and southern Sichuan, southeastern Tibet, northern and central Yunnan and their surrounding areas. The largest area of highly suitable habitat occurs in Sichuan and Yunnan and covers an area of approximately 0.094 × 10^6^ km^2^, accounting for 87% of the highly suitable habitats in China.

### Suitable habitat distributions under global warming scenarios

**Figure 3:**
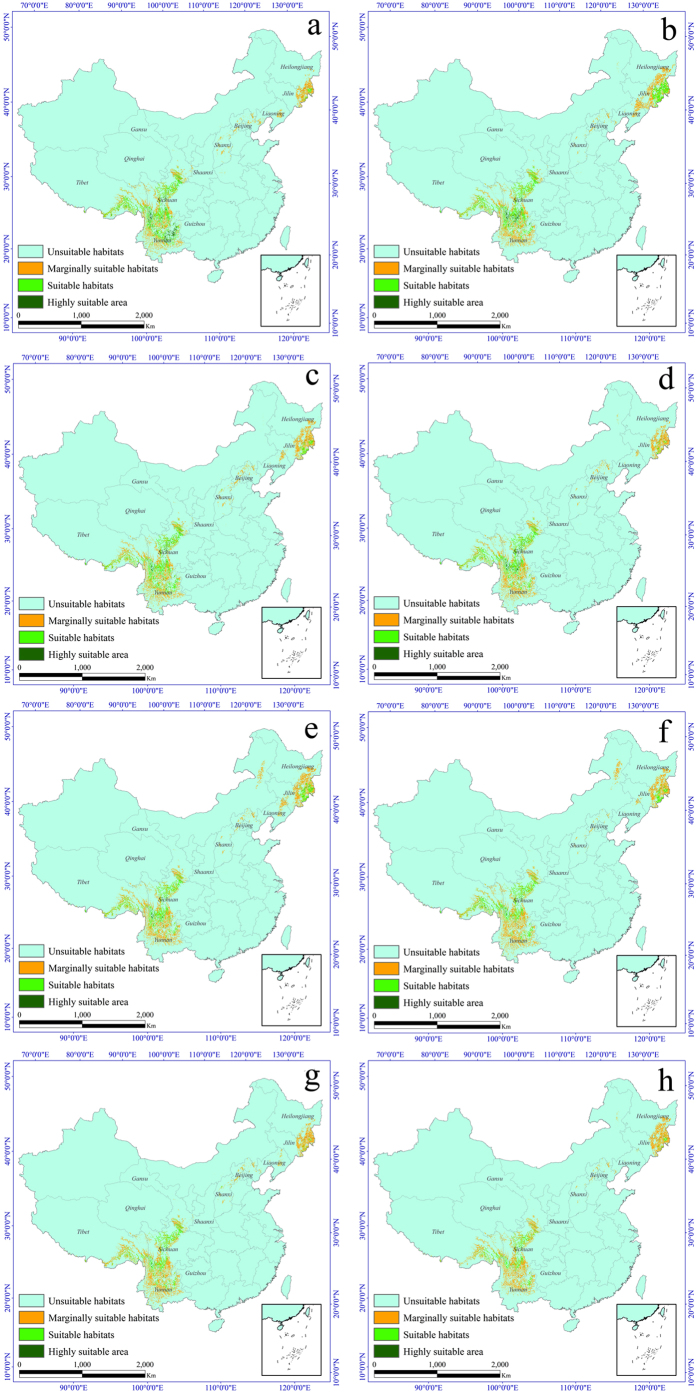
Distribution of varying habitat suitability for *Tricholoma matsutake* under different climate change scenarios in China. (**a**) 2050s in the RCP 2.6 climate scenario. (**b**) 2070s in the RCP 2.6 climate scenario. (**c**) 2050s in the RCP 4.5 climate scenario. (**d**) 2070s in the RCP 4.5 climate scenario. (**e**) 2050s in the RCP 6.0 climate scenario. (**f**) 2070s in the RCP 6.0 climate scenario. (**g**) 2050s in the RCP 8.5 climate scenario. (**h**) 2070s in the RCP 8.5 climate scenario. All maps were plotted using ArcGIS 9.3 (ESRI, Redlands, CA, USA, http://www.esri.com/).

**Figure 4:**
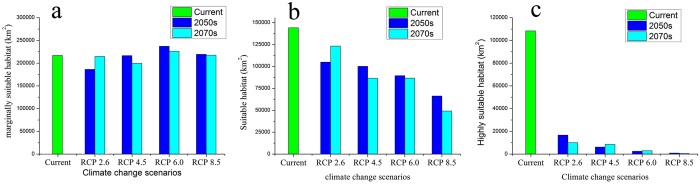
Area of varying habitat suitability of *Tricholoma matsutake* under different climate change scenarios in China. (**a**) Marginally suitable habitats for *T. matsutake*. (**b**) Suitable habitats for *T. matsutake*. (**c**) Highly suitable habitats for *T. matsutake*.

Using the projected bioclimatic variables and the same topographic, soil type, and vegetation type variables, we reassessed the current *T. matsutake* distribution to reflect a changing climate. These predictions provide a comprehensive assessment of *T. matsutake* habitat suitability. Using the same standard of classification, the future habitat suitability for *T. matsutake* was classified into four classes. The final results were derived from the average of the three general circulation models (GCMs). The model forecast (Figs 3 and 4) indicates that under all four climate change scenarios and for both periods, the area of marginally suitable habitat of *T. matsutake* is relatively stable. The area varies from 0.19 × 10^6^ km^2^ (under RCP 2.5 in the 2050s) to 0.24 × 10^6^ km^2^ (under RCP 6.0 in the 2050s). The area of suitable habitat of *T. matsutake* decreases gradually under all four climate change scenarios. By the 2050s, the area of suitable habitat of *T. matsutake* under the RCP 8.5, RCP 6.0, RCP 4.5 and RCP 2.6 scenarios is 0.066 × 10^6^ km^2^, 0.089 × 10^6^ km^2^, 0.10 × 10^6^ km^2^, and 0.11 × 10^6^ km^2^, respectively. By the 2070s, the area of suitable habitat of *T. matsutake* continues to decline. This tendency is pronounced under the RCP 8.5 scenario. Under this scenario, the area of suitable habitat of *T. matsutake* is 0.049 × 10^6^ km^2^, only one-third of its original size. The suitable habitat area in central Yunnan also decreases significantly. Similar decreases are predicted in southeastern Heilongjiang and northeastern Jilin, along with increased habitat fragmentation. The highly suitable habitats for *T. matsutake* significantly decline and nearly disappear under all four climate change scenarios. By the 2050s, only 0.93 × 10^3^ km^2^, 2.5 × 10^3^ km^2^, 6.2 × 10^3^ km^2^, and 16.7 × 10^3^ km^2^ of the highly suitable habitat of *T. matsutake* remain under the RCP 8.5, RCP 6.0, RCP 4.5 and RCP 2.6 scenarios, respectively. By the 2070s, the highly suitable habitat of *T. matsutake* in China decreases sharply, and only a small area in northwestern Yunnan, central Sichuan, and southeastern Tibet remains.

## Discussion

### The rationality of the model

The distinctive life cycles and growth forms of fungi pose unique challenges for mapping fungal species distributions at large spatial and temporal scales^15^. SDMs provide a possible solution to this problem. The 19 bioclimatic variables used in this research were derived from monthly temperature and rainfall values, which capture annual ranges, seasonality, and limiting environmental factors. Compared to monthly temperature and rainfall values, bioclimatic variables are more biologically meaningful. Furthermore, these data have been proven to be useful for SDMs and are among the most widely used data sets^35^. In contrast to plant species, fungal species, such as *T. matsutake*, will not grow in the absence of suitable vegetation and soil conditions, even if climate and topography are favorable. Thus, soil type and vegetation type variables should be considered limiting factors during the modeling process. Because of their specific growth requirements, fungal species are considered vulnerable to climate change.

MaxEnt originated from maximum entropy theory and is one of the most popular tools for species distribution modeling^32^. MaxEnt focuses on fitting a probability distribution for the occurrence of a species to the set of pixels across the study region^31^. The theoretical basis for MaxEnt is that for appropriate constraints, the best explanation for unknown phenomena will maximize the entropy of the probability distribution. When modeling species distribution, these constraints consist of the values of those pixels where the species occurs. MaxEnt typically outperforms other methods in terms of predictive accuracy^32^.

### Features of *T. matsutake* range shifts under global warming scenarios in China

The model results indicated that the range shifts of the two main regions of *T. matsutake* distribution have different features. In northeastern China, the suitable habitat of *T. matsutake* continues to decrease in are and move north, and marginal habitat is gradually increased, with the boundary shifted northward. In southwestern China, both the suitable and highly suitable habitat of *T. matsutake* decreases extensively, although the northern boundary does not show obvious northward movement. The reasons for his lack of northward movement are as follows. First, the northern boundary of the *T. matsutake* distribution in this area is along the edge of the Qinghai-Tibet Plateau, and new suitable habitat cannot form due to the dramatic elevation changes, complicated topography and variable climate. Second, in contrast to plant species, fungal species such as *T. matsutake* will not grow in the absence of suitable vegetation and soil conditions, even if climate and topography are favorable. Thus, soil type and vegetation type variables should be considered limiting factors during the modeling process. As global warming increases, the distribution of *T. matsutake* theoretically shifts poleward and to higher elevations. However, vegetation succession and the formation of suitable soil occur slowly in these areas under natural conditions, hindering the ability of fungal species to shift their ranges. Third, the shift of *T. matsutake* habitat might be constrained by the movement of its hosts. Using the MaxEnt method, we simulated the future distributions of *T. matsutake* host trees (*Pinus densata, Pinus yunnanensis*, and *Pinus densiflora*) in the 2050s and 2070s. The results showed that the northern boundary of the distribution of host trees also show no obvious trend of northward movement (see Supplementary materials).

### The dominant variables’ response to suitability

According to Suz *et al*.^15^, the main gaps in current knowledge of ectomycorrhizal (ECM) fungi include the environmental and ecological factors that control mycorrhizal distributions at the landscape and larger scales. MaxEnt provides a practical method to solve this problem. Using an iterative method, MaxEnt determines the relative contributions of environmental variables to species distribution. With each iteration of the training algorithm, the increase in regularized gain is added to the contribution of the corresponding variable and vice versa^31^,^32^. In this research, the estimates of the relative contributions of the environmental variables indicate that bio12 (Annual Precipitation), bio3 (Isothermally), and bio2 (Mean Diurnal Range) are the key factors that determine the distribution of *T. matsutake*, with contribution rates of 33.8%, 29.9%, and 10.9%, respectively. In addition, the bio10 (Mean Temperature of Warmest Quarter), bio1 (Annual Mean Temperature), bio5 (Max Temperature of Warmest Month), bio18 (Precipitation of Warmest Quarter), elevation, and slope variables had relatively high contributions to the distribution of *T. matsutake.*

**Figure 5:**
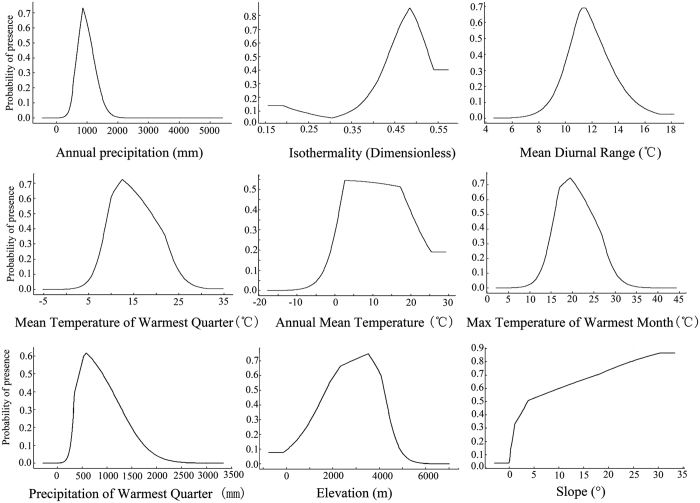
Probability relationships between dominant climate factors and geographic distribution of *Tricholoma matsutake*.

To further elucidate the features of climate and geography that created the suitable habitat of *T. matsutake*, we created different MaxEnt models using only one of the corresponding variables mentioned above. According to the quantitative relationship between environmental variables and habitat suitability (logistic probability of presence), we created response curves that illustrate how the logistic prediction changes as each environmental variable changes (Fig. 5). According to the response curves, we calculated the suitable ranges of environmental variables for the distribution area of *T. matsutake* (logistic probability of presence >0.3). The ranges are 600–1300 mm for bio12, greater than 0.41 for bio3, 10–13.5 °C for bio2, 7–23 °C for bio10, 0–22 °C for bio1, 14–27.5 °C for bio5, 400–1300 mm for bio18, 1000–4400 m for elevation, and greater than 3 degrees for slope. The logistic probability of presence increased gradually as the slope increased.

Yuan *et al*.^36^ produced a preliminary summary of the climate environment characteristics in *T. matsutake*-producing areas in the Hengduan Mountains in southwestern China. This study reported that *T. matsutake* mainly grows at altitudes between 3600 and 4000 m and on steep slopes. These areas are in humid or semi–humid parts of the alpine cold temperate zone or the sub–high mountain cold temperate zone, with a clear distinction between the dry season and rainy season. The annual precipitation is 800–1000 mm, with a smaller annual range of temperature and a larger diurnal temperature range. *T. matsutake* growth occurs in the rainy season, and the yields depend mainly on the amount of rainfall from June to August. During this period, *T. matsutake* grows normally and has high productivity if the monthly rainfall depth is 100–200 mm. Conversely, severe and persistent drought from June to August will decrease *T. matsutake* production and quality. During the growth phase, large temperature differences between day and night are a key factor for the growth and development of *T. matsutake*. During this time, the temperature should be 18–25 °C during the daytime and lower at night. Higher diurnal temperature variations will promote mycelial proliferation, differentiation, and fruiting during the squaring period. This record is consistent with the results of our model and further confirms the scientific and practical rationale of the model.

### The shifts of the suitable habitat of *T. matsutake* in other Asian countries

**Figure 6:**
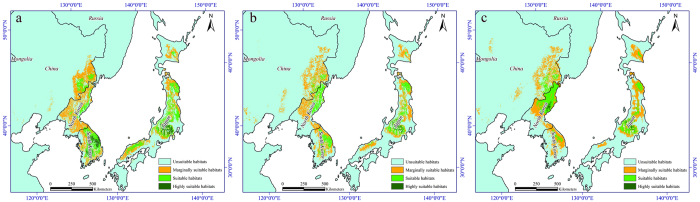
Distribution of varying habitat suitability for *Tricholoma matsutake* at different times in other Asian countries. (**a**) Current distribution. (**b**) 2050s distribution. (**c**) 2070s distribution. All maps were plotted using ArcGIS 9.3 (ESRI, Redlands, CA, USA, http://www.esri.com/).

To further elucidate the features of shifts in the suitable habitat of *T. matsutake* in Asia, particularly in traditional *T. matsutake* production areas, we expanded the scope of the study area to include North Korea, South Korea, Japan and areas of Russia that border northeast China. We added 26 occurrence data of *T. matsutake* in these areas, and we obtained soil type variable data from the Harmonized World Soil Database (HWSD, http://globalchange.nsdc.cn) and vegetation type variable data from GlobCover 2009 (Global Land Cover Map, http://www.fao.org/home/en/). We then used the same method as applied previously to simulate the influence of climate change on the potential geographic distribution of *T. matsutake* in these areas. The final results were derived from the average of the three general circulation models (GCMs) and four IPCC–CMIP5 representative concentration pathways (Fig. 6).

The model forecast indicated that at present, the highly suitable habitat of *T. matsutake* is mainly located in east-central South Korea and central Japan. By the 2050s, highly suitable habitat has largely disappeared; in Japan, it is extensively reduced and shifted northward. By the 2070s, highly suitable habitat appears in east-central North Korea, and in Japan, it continues to decrease and move north. Over time, the suitable habitat of *T. matsutake* in South Korea and Japan declines sharply and shifts to high-latitude areas, whereas in North Korea, the range of suitable habitat gradually increases. The marginal habitat of *T. matsutake* under climate change in the whole area remains largely stable, with the boundary shifted northward. By the 2070s, little area of marginal habitat appears in Russia.

### Other factors that affect the distribution of *T. matsutake*

In addition to climate change, human interference is an important factor that influences the distribution of *T. matsutake*. Because of its medicinal effects and attractive flavor, *T. matsutake* is commercially important in Asia as a valuable food^18^. As the popularity of this mushroom increases in both the domestic and international food markets, wild resources of *T. matsutake* are being depleted. Theoretically, *T. matsutake* can be distributed in all highly suitable habitats; however, due to anthropogenic effects, such as over–grazing and excessive deforestation, some traditional habitats of *T. matsutake* no longer host this species, and the phenomenon of habitat fragmentation is irreversible. Many efforts have been made to establish an artificial cultivation system for high–quality *T. matsutake*; however, almost all attempts have failed^22^,^23^. Therefore, *T. matsutake* can be conserved only by maintaining and increasing natural production in highly suitable habitats. Fortunately, with increasing societal interest in environmental protection and the enactment of relevant laws and regulations, *T. matsutake* shiroes are increasingly protected, especially in nature reserves. In addition, artificial introductions into appropriate wild environments have expanded the native habitat of this species.

The natural reproduction ability of *T. matsutake* is also a key factor affecting its distribution. As an ECM fungus, the reproductive characteristics of *T. matsutake* are similar to those of other ECM fungi in nature. The species surrounds living plant rootlets, has a strong host preference, and expands its populations by dispersal of basidiospores, fragmented mycelia, mitotic sporulation, and sclerotial and extraradical mycelial extension^15^,^18^. *T. matsutake* has more stringent environmental requirements for its reproduction, particularly precipitation and temperature^18^,^37^. Therefore, the continuous development of global warming will affect the natural reproduction ability of *T. matsutake* in traditional habitats, further aggravating the influence of climate change on *T. matsutake* distribution.

## Methods

### Species occurrence data collection

All occurrence data of *T. matsutake* were obtained from the published scientific literature and field survey reports^17^,^20^,^21^,^38^,^39^,^40^,^41^,^42^,^43^,^44^ and represent fruiting body data. To perform detailed MaxEnt models, we chose only the data with precise location information to ensure improved geographic accuracy. After removing duplicate points, a total of 91 species presence locations were used for analysis (Fig. 1).

### Climatic data and environmental variables

According to the growth conditions of *T. matsutake* as well as the operability and feasibility of the data, four datasets including 19 bioclimatic variables, three topographic variables, soil type variables, and vegetation type variables were chosen (Table 1) for this study. The bioclimatic variables were downloaded from the WorldClim database. These variables were generated using averaged interpolated climate data from 1950 to 2000^45^ at a resolution of 30″ (approximately 1 km^2^). The potential values for bioclimatic variables under future climate conditions in the 2050s (average for 2041–2060) and 2070s (average for 2061–2080) were derived from three GCMs (CCSM4, MIROC5, and BCC–CSM1–1) under four IPCC–CMIP5 representative concentration pathways (RCP: RCP 8.5, RCP 6, RCP 4.5 and RCP 2.6). The bioclimatic variables for the future scenarios were obtained from the International Centre for Tropical Agriculture (http://ccafs–climate.org), at a resolution of 30″ (approximately 1 km^2^).

Topographic variables including elevation, slope and aspect are important factors for the distribution patterns of vegetation. Elevation variables at 30″ (approximately 1 km^2^) resolution were downloaded from the WorldClim database. The ArcGIS spatial analysis function was used to obtain the slope and aspect variables from the elevation data. The source of the soil type variables was the 1:1 million soil database of China; the data set was provided by the Data Center for Resources and Environmental Sciences, Chinese Academy of Sciences (RESDC) (http://www.resdc.cn). The vegetation type variables were obtained from the 1:1 million China vegetation data set provided by the Environmental and Ecological Science Data Center for West China, National Natural Science Foundation of China (http://westdc.westgis.ac.cn)^46^,^47^. Both the vegetation type and soil type variables had a resolution of 30″ (approximately 1 km^2^). In this study, we assumed that the topographic, soil type and vegetation type variables remain unchanged over the next 70 years.

### Model evaluation

In this study, the climatic and environmental variables were divided into two categories. The first category used for MaxEnt modeling included the 19 bioclimatic variables and three topographic variables. The second category included the limiting ecological variables, soil and vegetation type, both of which are categorical data.

Using the 19 bioclimatic variables, 3 topographic variables, and the *T. matsutake* occurrence data, we used MaxEnt software (MaxEnt version 3.3.3, http://www.cs.princeton.edu/~schapire/maxent/) to predict the current and future distribution of *T. matsutake*. MaxEnt has been extensively adapted to predict species range shifts under climate change and is based on the maximum entropy modeling technique. During the modeling procedure, 75% of the occurrence data were used for model calibration, and the remaining 25% of the data were used to test the predictive ability of the model. To guarantee the accuracy of the model, we performed 10 replications, and cross validation was maintained in the replicate runs. The AUC was used. The AUC ranges from 0.5 (random) to 1.0 (perfect discrimination), and values >0.8 indicate satisfactory performance^31^,^32^.

*T. matsutake* is a forest ectomycorrhizal fungus and is predominantly associated with coniferous trees in northeastern China but has been found in broad–leaved forests consisting of *Castanopsis* spp., *Quercus* spp. and alpine shrubs^20^,^21^,^36^,^42^,^43^. Therefore, the type of vegetation-environment suitable for *T. matsutake* is precisely known, and we defined two habitat suitability values for the vegetation type variable. A suitability value of 1 was used for coniferous, mixed coniferous, bushwood, and broad–leaved forests, and a suitability value of 0 was used for all other vegetation types. Because there are no explicit soil conditions for the growth of *T. matsutake*, we created a MaxEnt model that used only the soil type variables to assess the suitable requirements. Therefore, the distribution of comprehensive habitat suitability for *T. matsutake* was defined as follows [equation (1)]:


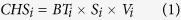


where *CHS*_*i*_ is the comprehensive value assessment of *T. matsutake* habitat suitability in each evaluation unit; *BT*_*i*_ is the value of the MaxEnt result based on 19 bioclimatic variables and 3 topographic variables in each evaluation unit; *S*_*i*_ is the result of the MaxEnt model that used only the soil type variables; and *V*_*i*_ is the habitat suitability value for vegetation. The range of *CHS*_*i*_ was defined as 0–1. We applied spatial analysis using ArcGIS 10.1 to create the raster and map the habitat suitability assessment with a resolution of 30″.

We used the model mentioned above to study the potential changes in the distributions of *T. matsutake*. The future scenarios were regionalized for the study area under four RCPs: RCP 8.5, RCP 6.0, RCP 4.5 and RCP 2.6, and two periods (the 2050s and 2070s). We ran separate models for each period using future bioclimatic variable data from the three GCMs and topographic variables. The future potential habitat predictions for *T. matsutake* under each IPCC–CMIP5 RCP and for both periods were obtained by averaging the results. For further analysis, we classified the comprehensive value of assessment of *T. matsutake* habitat suitability into four classes: unsuitable habitats (*CHS*_*i*_ < 0.3), marginally suitable habitats (0.3 ≤ *CHS*_*i*_ < 0.5), suitable habitats (0.5 ≤ *CHS*_*i*_ < 0.7), and highly suitable habitats (*CHS*_*i*_ ≥ 0.7). We mapped the comprehensive distribution of habitat suitability of *T. matsutake* in China (Fig. 2) and calculated the area of all habitat suitability grades.

## Additional Information

**How to cite this article**: Guo, Y. *et al*. Prediction of the potential geographic distribution of the ectomycorrhizal mushroom *Tricholoma matsutake* under multiple climate change scenarios. *Sci. Rep.* **7**, 46221; doi: 10.1038/srep46221 (2017).

**Publisher's note:** Springer Nature remains neutral with regard to jurisdictional claims in published maps and institutional affiliations.

## Supplementary Material

Supplementary Information

## Figures and Tables

**Table 1:** Environmental variables used for predicting the potential geographic distribution of *Tricholoma matsutake* in China.

Environmental index	Code	Description	Unit	Source
Climatic variables	Bio1	Annual mean air temperature	Degrees Celsius	http://www.worldclim.org/
	Bio2	Mean diurnal temperature range	Degrees Celsius	http://www.worldclim.org/
	Bio3	Isothermality	Dimensionless	http://www.worldclim.org/
	Bio4	Temperature seasonality	Degrees Celsius	http://www.worldclim.org/
	Bio5	Max temperature of warmest month	Degrees Celsius	http://www.worldclim.org/
	Bio6	Min temperature of coldest month	Degrees Celsius	http://www.worldclim.org/
	Bio7	Temperature annual range	Degrees Celsius	http://www.worldclim.org/
	Bio8	Mean temperature of wettest quarter	Degrees Celsius	http://www.worldclim.org/
	Bio9	Mean temperature of driest quarter	Dimensionless	http://www.worldclim.org/
	Bio10	Mean temperature of warmest quarter	Degrees Celsius	http://www.worldclim.org/
	Bio11	Mean temperature of coldest quarter	Degrees Celsius	http://www.worldclim.org/
	Bio12	Annual precipitation	Millimeters	http://www.worldclim.org/
	Bio13	Precipitation of wettest month	Millimeters	http://www.worldclim.org/
	Bio14	Precipitation of driest month	Millimeters	http://www.worldclim.org/
	Bio15	Precipitation seasonality	Fraction	http://www.worldclim.org/
	Bio16	Precipitation of wettest quarter	Millimeters	http://www.worldclim.org/
	Bio17	Precipitation of driest quarter	Millimeters	http://www.worldclim.org/
	Bio18	Precipitation of warmest quarter	Millimeters	http://www.worldclim.org/
	Bio19	Precipitation of coldest quarter	Millimeters	http://www.worldclim.org/
Topographic variables	ASL	Elevation above sea level	Meters	http://www.worldclim.org/
	SLOP	Slope	Degree	
	ASPE	Aspect	Degree	
Soil type variables	soil	Soil type		http://www.resdc.cn
Vegetation type variables	vegetation	Vegetation type		http://westdc.westgis.ac.cn
