# Supplementary material for: Prediction of the potential geographic distribution of the ectomycorrhizal mushroom Tricholoma matsutake under multiple climate change scenarios
Source: Sci Rep. 2017 Apr 10;7:46221. doi: 10.1038/srep46221 (PMC5385516; doi:10.1038/srep46221)
Supplement: Supplementary Information [file srep46221-s1.doc]

**Prediction of the potential geographic distribution of** **the ectomycorrhizal mushroom *Tricholoma matsutake* under multiple climate change scenarios**

Yanlong Guo^1,2^, Xin Li^1,3∗^, Zefang Zhao^4^, Haiyan Wei^4^, Bei Gao^4^, and Wei Gu^5^

^1^ Northwest Institute of Eco-Environment and Resources, Chinese Academy of Sciences, Lanzhou 730000, China

^2^ University of Chinese Academy of Sciences, Beijing 100049, China

^3^ CAS Center for Excellence in Tibetan Plateau Earth Sciences, Beijing 100101, China

^4^ College of Tourism and Environment, Shaanxi Normal University, Xian 710119 China

^5^ College of Life Sciences, Shaanxi Normal University, Xian 710119 China

***Corresponding author**: Xin Li, 320 West Dong Gang Road, Northwest Institute of Eco-Environment and Resources, Chinese Academy of Sciences, Lanzhou 730000, Gansu Province, China E-mail: lixin@lzb.ac.cn

**Supplementary materials**

This study used the MaxEnt method and 19 bioclimatic variables and three topographic variables to map the future distributions of *T. matsutake* host tree species (*Pinus densata*, *Pinus yunnanensis*, and *Pinus densiflora*) in the 2050s and 2070s. The final results were derived from the average of three general circulation models (GCMs) and four IPCC–CMPI5 representative concentration pathways. All occurrence data of the three host trees were obtained from The Global Biodiversity Information Facility (GBIF, http://www.gbif.org/).


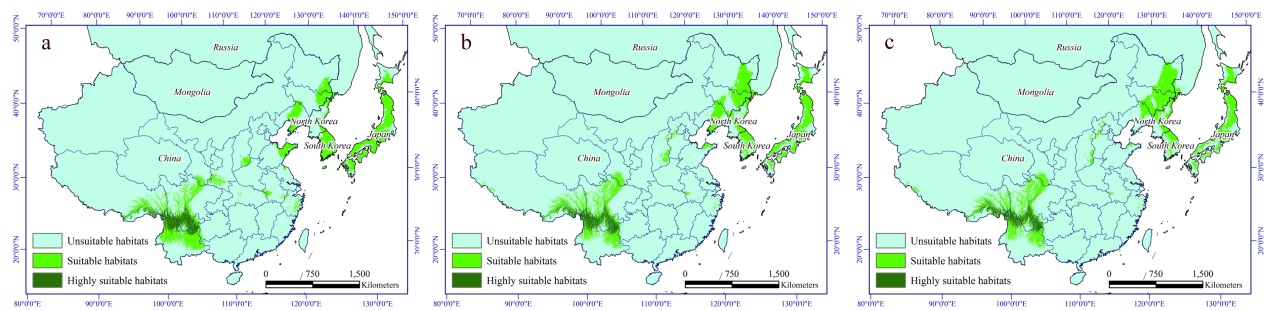


**Figure 1.** Distribution of habitat suitability for three main *T matsutake* host trees at different times. a, currently. b, 2050s. c, 2070s. All maps were plotted using ArcGIS 9.3 (ESRI, Redlands, CA, USA, http://www.esri.com/). "Unsuitable habitat" refers to area that is not suitable for any of the host tree species. "Suitable habitat" refers to area that is suitable for one of the three host tree species, and "Highly suitable habitat" refers to area that is suitable for two of three host tree species.


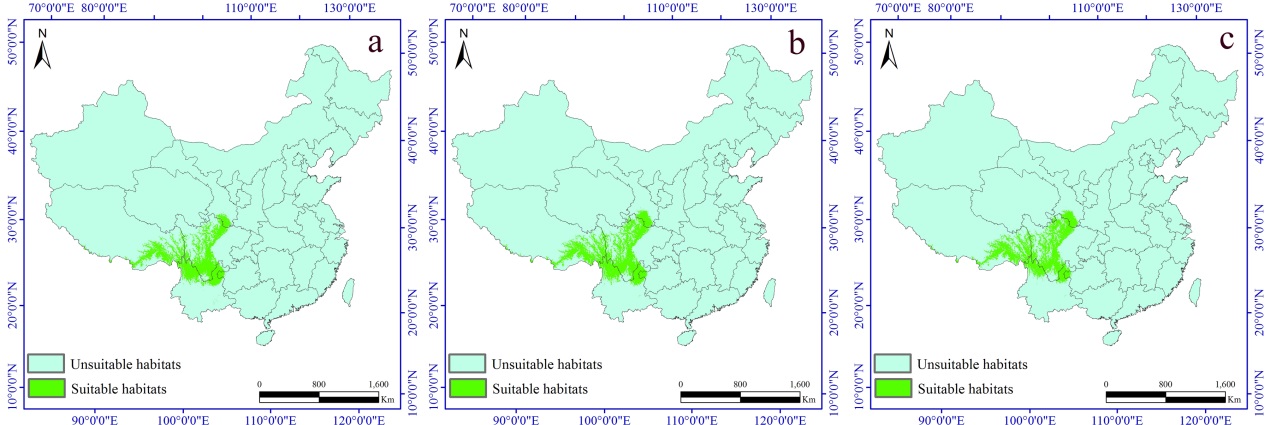


**Figure 2.** Distribution of habitat suitability for *Pinus densata* at different times. a, currently. b, 2050s. c, 2070s. All maps were plotted using ArcGIS 9.3 (ESRI, Redlands, CA, USA, <http://www.esri.com/>).


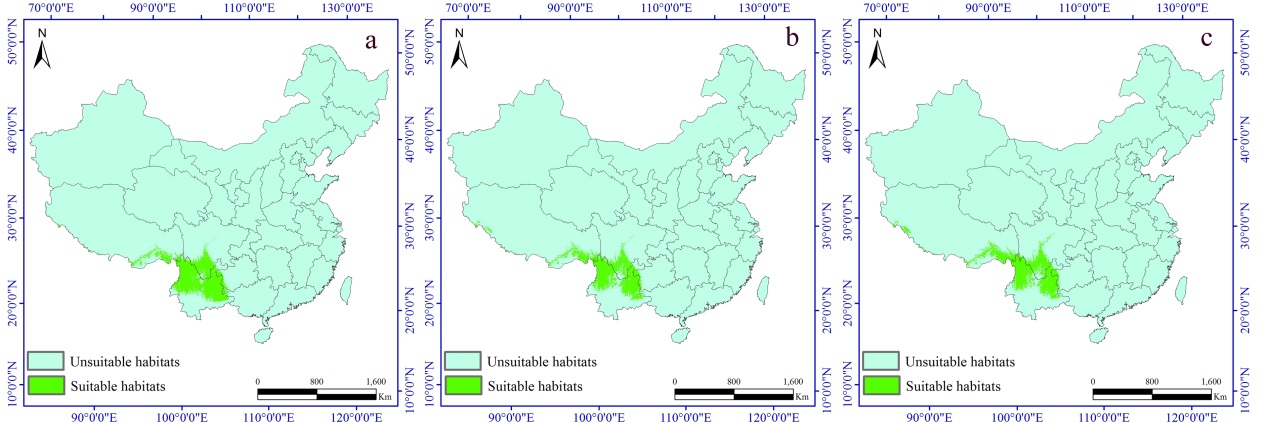


**Figure 3.** Distribution of habitat suitability for *Pinus yunnanensis* at different times. a, currently. b, 2050s. c, 2070s. All maps were plotted using ArcGIS 9.3 (ESRI, Redlands, CA, USA, <http://www.esri.com/>).


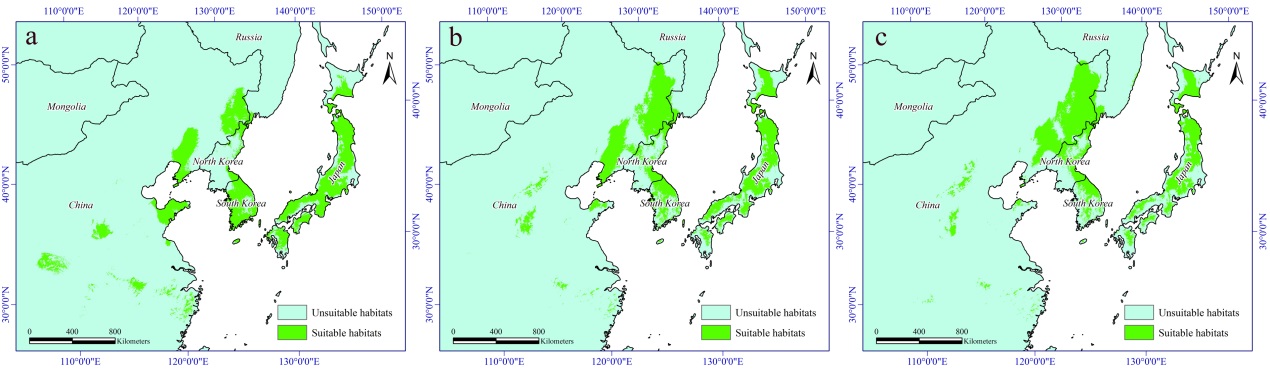


**Figure 4.** Distribution of habitat suitability for *Pinus densiflora* at different times. a, currently. b, 2050s. c, 2070s. All maps were plotted using ArcGIS 9.3 (ESRI, Redlands, CA, USA, <http://www.esri.com/>).
